# Sleep Disordered Breathing, Obesity and Atrial Fibrillation: A Mendelian Randomisation Study

**DOI:** 10.3390/genes13010104

**Published:** 2022-01-02

**Authors:** Maddalena Ardissino, Rohin K. Reddy, Eric A. W. Slob, Kiran H. K. Patel, David K. Ryan, Dipender Gill, Fu Siong Ng

**Affiliations:** 1 National Heart and Lung Institute, Imperial College London, London W12 0NN, UK; maddalena.ardissino13@imperial.ac.uk (M.A.); rohin.reddy17@imperial.ac.uk (R.K.R.); kiran.patel@imperial.ac.uk (K.H.K.P.); 2 Nuffield Department of Population Health, Old Road Campus, University of Oxford, Oxford OX3 7LF, UK; 3 MRC Biostatistics Unit, School of Clinical Medicine, University of Cambridge, Cambridge CB2 0SR, UK; eric.slob@mrc-bsu.cam.ac.uk; 4 Clinical Pharmacology Group, Pharmacy and Medicines Directorate, St George’s University Hospitals NHS Foundation Trust, London SW17 0QT, UK; davidkdryan@gmail.com (D.K.R.); dipender.gill@imperial.ac.uk (D.G.); 5 Clinical Pharmacology and Therapeutics Section, Institute for Infection and Immunity, St George’s, University of London, London SW17 0RE, UK; 6 Department of Epidemiology and Biostatistics, School of Public Health, Imperial College London, London W2 1PG, UK; 7 Novo Nordisk Research Centre Oxford, Old Road Campus, Oxford OX3 7FZ, UK

**Keywords:** atrial fibrillation, sleep-disordered breathing, obstructive sleep apnoea, obesity, Mendelian randomization

## Abstract

It remains unclear whether the association between obstructive sleep apnoea (OSA), a form of sleep-disordered breathing (SDB), and atrial fibrillation (AF) is causal or mediated by shared co-morbidities such as obesity. Existing observational studies are conflicting and limited by confounding and reverse causality. We performed Mendelian randomisation (MR) to investigate the causal relationships between SDB, body mass index (BMI) and AF. Single-nucleotide polymorphisms associated with SDB (*n* = 29) and BMI (*n* = 453) were selected as instrumental variables to investigate the effects of SDB and BMI on AF, using genetic association data on 55,114 AF cases and 482,295 controls. Primary analysis was conducted using inverse-variance weighted MR. Higher genetically predicted SDB and BMI were associated with increased risk of AF (OR per log OR increase in snoring liability 2.09 (95% CI 1.10–3.98), *p* = 0.03; OR per 1-SD increase in BMI 1.33 (95% CI 1.24–1.42), *p* < 0.001). The association between SDB and AF was not observed in sensitivity analyses, whilst associations between BMI and AF remained consistent. Similarly, in multivariable MR, SDB was not associated with AF after adjusting for BMI (OR 0.68 (95% CI 0.42–1.10), *p* = 0.12). Higher BMI remained associated with increased risk of AF after adjusting for OSA (OR 1.40 (95% CI 1.30–1.51), *p* < 0.001). Elevated BMI appears causal for AF, independent of SDB. Our data suggest that the association between SDB, in general, and AF is attributable to mediation or confounding from obesity, though we cannot exclude that more severe SDB phenotypes (i.e., OSA) are causal for AF.

## 1. Introduction

Current clinical guidelines support an integrated approach to atrial fibrillation (AF) management, encompassing anticoagulation, symptom management and risk factor optimisation, with recent focus on obesity and obstructive sleep apnoea (OSA) as two reversible risk factors [1]. Sleep-disordered breathing (SDB) is a general term describing difficulties in breathing during sleep ranging from snoring to OSA, which is increasing in prevalence [2,3]. OSA is the most common form of SDB in the general population, characterised by hypopnoeic and apnoeic collapses of the upper airway during sleep despite ongoing respiratory effort [2]. Based on several observational studies showing an association between OSA with higher rates of AF [4,5,6] and observational meta-analysis associating continuous positive airway pressure (CPAP) treatment with reductions in AF recurrence [7], previous clinical guidelines for AF contained a Class IIa recommendation that OSA treatment should be optimised to reduce AF recurrence and improve treatment [1].

However, more recent clinical guidelines have downgraded that recommendation in favour of a Class IIb recommendation that OSA management may be considered to reduce AF incidence, progression, recurrence and symptoms [1]. This reflects a subsequent appreciation of the uncertainty in the evidence base due to the dearth of randomised controlled trial (RCT) evidence and the lack of consensus from observational data. Some large observational studies have failed to demonstrate an association between OSA and AF that is independent of other cardiovascular risk factors [8,9]. Furthermore, the presence of OSA, and its treatment with CPAP, have recently been shown not to impact arrhythmia outcomes following ablation [10,11] or cardioversion [10]. Early and small RCTs have shown no impact of CPAP on time to AF recurrence post-cardioversion [12] or differences in AF burden or quality of life [13].

Complicating interpretation of the data on SDB/OSA and AF is the strong association of both SDB/OSA and AF with obesity, which may act as a confounder. Obesity has been shown to be associated with AF [14], with the effects thought to be mediated in part by electrotonic and paracrine effects of epicardial fat [15]. Obesity and SDB are also closely related, whereby obesity both predisposes to, and potentiates SDB [16,17], and developing SDB is associated with subsequent weight gain [18]. With currently available retrospective observational data, it is difficult to determine whether the relationship between SDB/OSA and AF is mediated or confounded through obesity and raised body mass index (BMI), or whether OSA causes AF directly through independent pathways.

Mendelian randomisation (MR) utilises genetic variants in instrumental variable analysis to investigate relationships between modifiable risk factors and outcomes using observational data [19]. Leveraging genetic variants that are independently and randomly inherited as proxies for modifiable exposures allows for causal inference concerning outcomes. In this way, MR can potentially overcome limitations associated with classical observational epidemiology, namely confounding and reverse causality [19], and may permit delineation of complex pathways relating SDB, BMI and AF. A recent univariable MR study reported a causal association between five single-nucleotide polymorphisms (SNPs) associated with OSA and AF [20]. However, some of the employed OSA instruments were also associated with BMI or whole-body, fat-free mass at the genome-wide significance level, raising the question of whether this relationship was partially mediated via obesity. We therefore applied the multivariable MR paradigm to investigate the relationship between genetically predicted SDB and BMI on AF, and to explore the presence of genetic evidence supporting a direct causal effect of SDB in the development of AF independent of BMI.

## 2. Materials and Methods

### 2.1. Ethical Approval, Data Availability and Reporting

All data used for this study are publicly available and their original studies are cited. All these studies obtained relevant participant consent and ethical approval. The paper is reported on the basis of recommendations by The Strengthening the Reporting of Observational Studies in Epidemiology-Mendelian randomization (STROBE-MR) guidelines [21].

### 2.2. Data Sources

For the primary analyses, genetic association estimates for BMI were obtained from the Genetic Investigation of Anthropometric Traits (GIANT) consortium summary statistics [22] on patients of European ancestry. Genetic association estimates for snoring obtained from the GWAS performed by Campos et al. [23] on European ancestry individuals were used as a proxy for SDB in general. The trait with the highest genetic correlation with snoring was sleep apnoea (rG = 0.78, SE = 0.17, *p*-value = 3 × 10^−5^ (LDSC $\chi^{2}$-test)). These analyses suggest that the SNPs for snoring studied are an appropriate and robust surrogate for genetically proxied SDB, and, henceforth, we will refer to the snoring exposure as SDB. Genetic association estimates for AF were obtained from the GWAS by Roselli et al. [24] on 55,114 cases and 482,295 European controls. Summaries of population characteristics for each of these studies are available in the original publications.

### 2.3. Instrumental Variable Selection

To estimate the effect of genetically predicted probable SDB and BMI, respectively, on AF, SNPs were selected if they had been associated with snoring or BMI in the respective data source studies at genome-wide significance (*p* < 5 × 10^−8^). Furthermore, SNPs were selected if they were in pair-wise linkage disequilibrium (LD) with *r*^2^ 0.001. Clumping was performed using the TwoSample MR package in R [25]. This resulted in 29 genome-wide significant SNPs for SDB and 453 genome-wide significant SNPs for BMI.

### 2.4. Statistical Power

Statistical power calculations for MR analyses were conducted using the online tool “mRnd calculator” [26] to estimate the minimum effects that we had at least 80% power to detect.

### 2.5. Statistical Analysis

**Figure 1:**
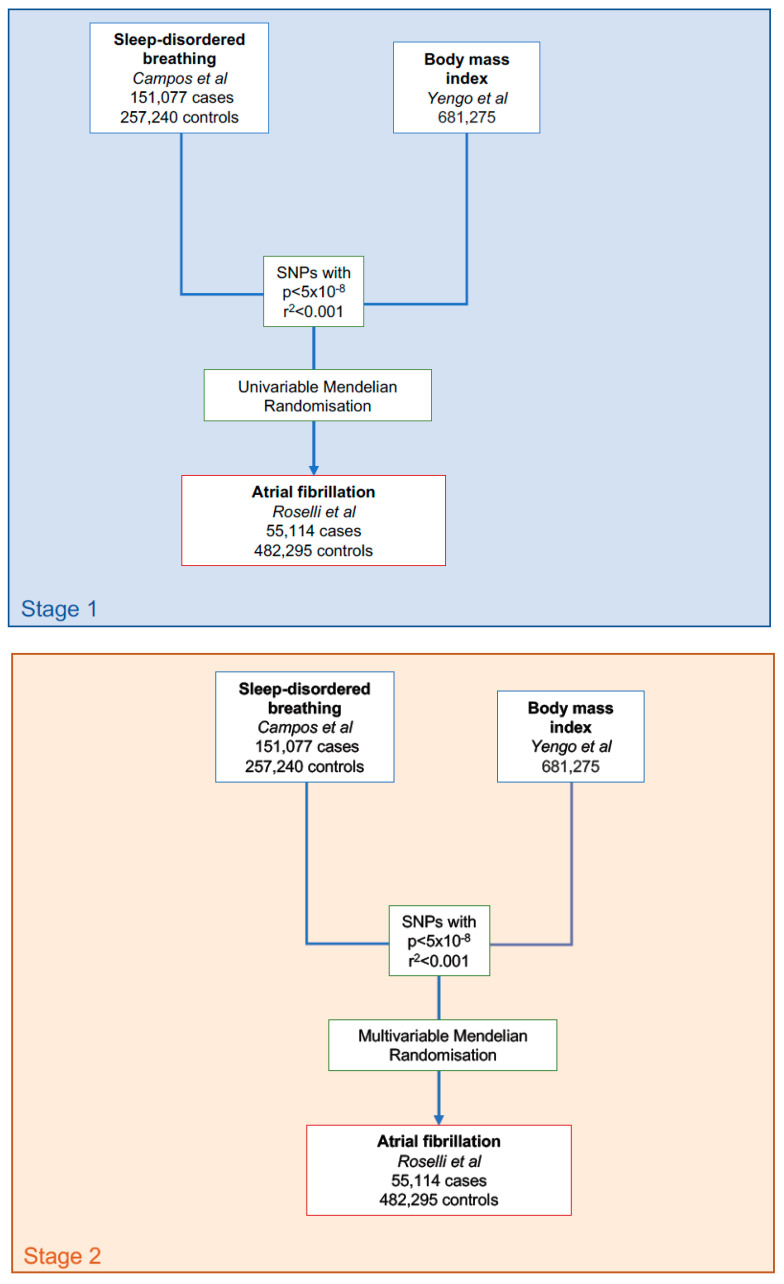
Data acquisition and statistical analysis flowchart.

The flowchart for the statistical analysis plan is displayed in Figure 1. Inverse-variance weighted (IVW) MR with multiplicative random effects was used as the primary analysis method [27]. The IVW MR approach assumes that genetic instruments for each risk factor satisfy the instrumental variable assumptions, which include the assumption that the instrumental variables are not associated with confounder traits of the association between the risk factor and the outcome of AF, and that the instrumental variables are only associated with AF through their association with the risk factor. The situation where, rather than acting solely through the genetically predicted risk factor of interest, genetic variants have effects on multiple risk factors influencing multiple parallel biological pathways and subsequent phenotypes is termed horizontal pleiotropy and constitutes an important potential violation of the instrumental variable assumptions [19].

To attempt to correct for any potential violations of the assumptions, we used MR-Egger regression [28], weighted median MR [29] and MR-PRESSO [30] as sensitivity analyses. We opted for these three analyses as they operate in different ways and rely on different assumptions for valid inferences to assess the reliability of MR analyses [31,32,33].

To investigate an effect of SDB on AF that is not mediated by BMI (and vice versa), summary data multivariable MR was performed. In this analysis, the variant-outcome genetic association estimates are regressed on the variant-exposure and variant-mediator estimates, weighted for the precision of the variant-outcome association, and with the intercept fixed to zero.

All analyses were performed using the MendelianRandomization [34] and TwoSample MR package in R version 4.0.4 [25]. M.A. had full access to all data in the study and takes responsibility for its integrity and data analysis.

## 3. Results

### 3.1. Statistical Power

The univariable MR analysis for BMI and AF had at least 80% power to detect AF ORs lower than 0.97 and higher than 1.04 per 1-SD kg/m^2^ increase in BMI. The univariable MR analysis for SDB and AF had at least 80% power to detect AF ORs lower than 0.97 and higher than 1.03 per log OR increase in genetically predicted OSA.

### 3.2. MR: SDB and AF

In IVW MR analysis, higher genetically predicted SDB was associated with increased risk of AF, OR 2.09 (95% CI 1.10–3.98, *p* = 0.03) per log OR increase in snoring liability. There was no significant evidence of pleiotropy (MR-Egger intercept 0.02, standard error (SE) 0.01, *p* = 0.20), but the significant association was no longer observed in MR-Egger (OR 0.24, 95% CI 0.01–6.53, *p* = 0.40), weighted median MR (OR 1.40, 95% CI 0.72–2.70, *p* = 0.32) analyses or MR-PRESSO (OR 1.85, 95% CI 1.00–3.41, *p* = 0.06, three SNPs excluded).

**Figure 2:**
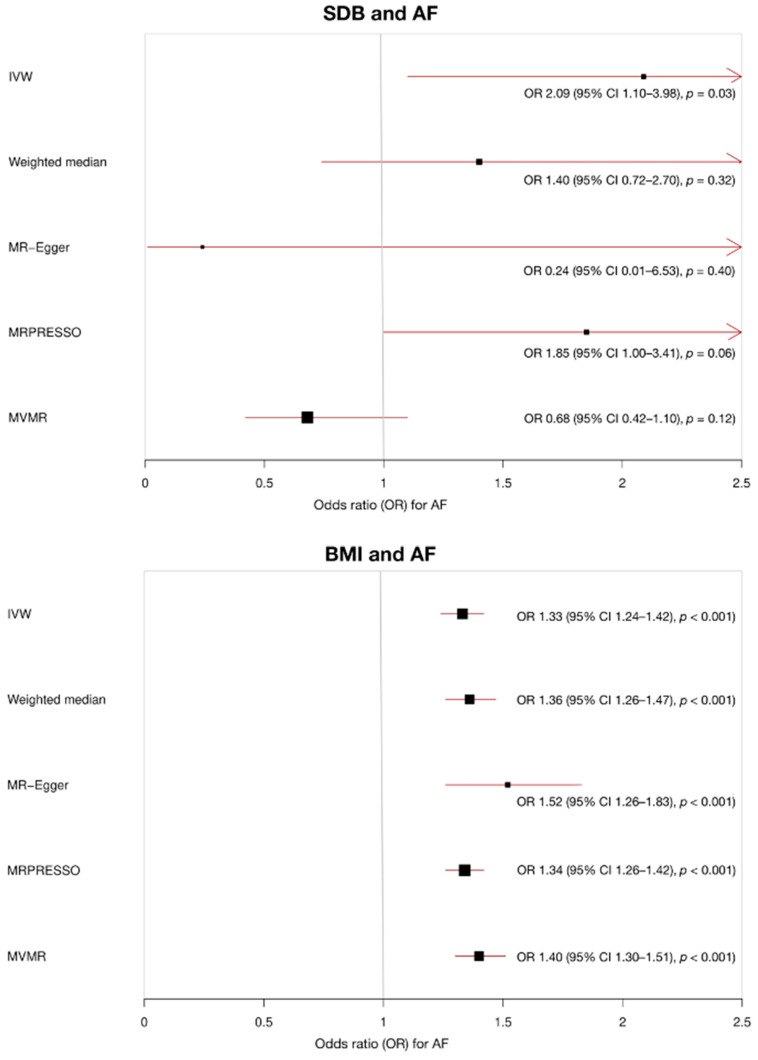
Mendelian randomisation (MR) results for the association of sleep-disordered breathing (SDB) and body mass index (BMI) with atrial fibrillation (AF). When SDB is the exposure (units of log odds ratio snoring liability), multivariable MR analysis adjusts for genetically predicted BMI. When BMI is the exposure (units of kg/m^2^), multivariable MR analysis adjusts for genetically predicted SDB.

**Figure 3:**
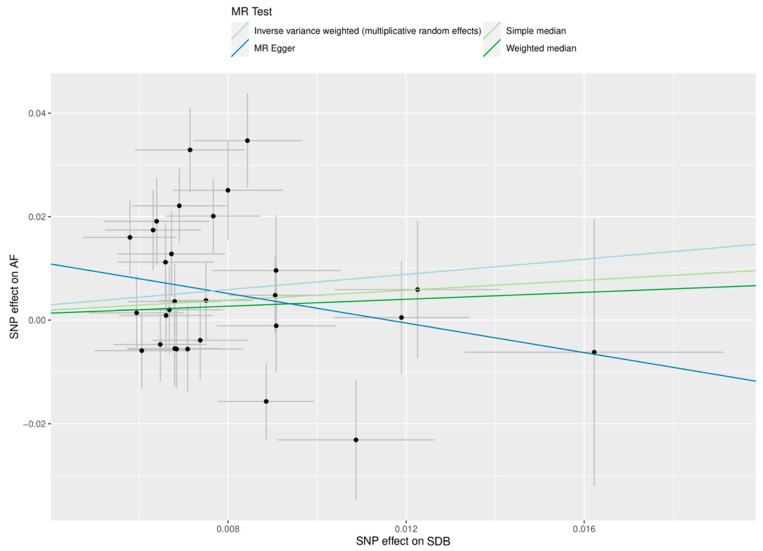
Mendelian randomisation (MR) scatter plot of genetic associations between sleep-disordered breathing (SDB) and atrial fibrillation (AF). The genetic association and corresponding 95% confidence interval (CI) for each instrumented single-nucleotide polymorphism (*n* = 29) with SDB (x-axis, units of log odds ratio snoring liability) and AF (y-axis, units of log odds ratio atrial fibrillation liability) are plotted. The gradient of each line represents the MR estimate for the corresponding model.

In multivariable MR analysis, genetically predicted SDB was not associated with AF after adjusting for genetically predicted BMI (OR 0.68, 95% CI 0.42–1.10, *p* = 0.12). The results are summarised in Figure 2 and Figure 3, with complete results displayed in Table S1. SNP MR estimates in the analysis of SDB and AF are displayed in Table S2.

### 3.3. MR: BMI and AF

There was consistent evidence of an association between genetically predicted BMI and AF across the IVW, MR-Egger and weighted median MR analyses. For each 1-SD kg/m^2^ increase in genetically predicted BMI, the MR IVW estimate identified increased risk of AF, OR 1.33 (95% CI 1.24–1.42, *p* < 0.001). There was no evidence of pleiotropy (MR-Egger intercept −2 × 10^−3^, SE 1 × 10^−3^, *p* = 0.14), and the association estimate remained consistent in MR-Egger (OR 1.52, 95% CI 1.26–1.83, *p* < 0.001), weighted median MR (OR 1.36, 95% CI 1.26–1.47, *p <* 0.001) and MR-PRESSO (OR 1.34, 95% CI 1.26–1.42, *p* < 0.001, 10 SNPs excluded) sensitivity analyses.

**Figure 4:**
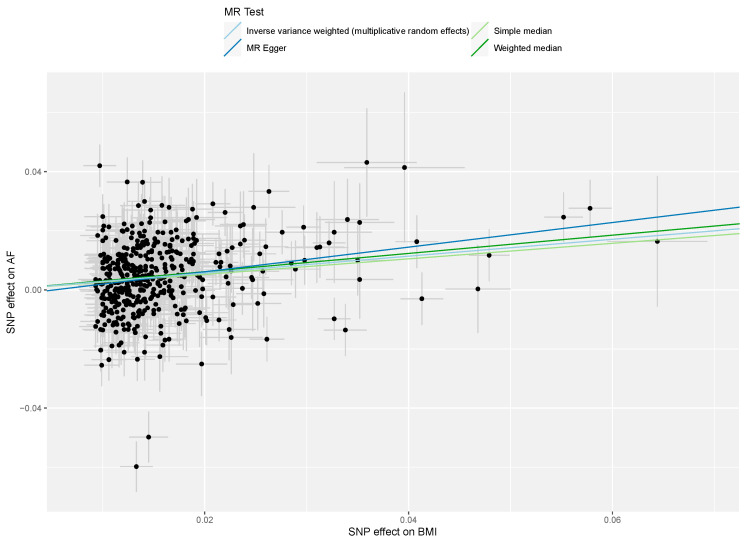
Mendelian randomisation (MR) scatter plot of genetic associations between body mass index (BMI) and atrial fibrillation (AF). The genetic association and corresponding 95% confidence interval (CI) for each instrumented single-nucleotide polymorphism (*n* = 453) with BMI (x-axis, units of kg/m^2^) and AF (y-axis, units of log odds ratio atrial fibrillation liability) are plotted. The gradient of each line represents the MR estimate for the corresponding model.

Multivariable MR identified an association between genetically predicted BMI and AF even after adjusting for genetically predicted SDB (OR 1.40, 95% CI 1.30–1.51, *p* < 0.001). The results are summarised in Figure 2 and Figure 4, with complete results displayed in Table S1. SNP MR estimates in the analysis of BMI and AF are displayed in Table S3.

## 4. Discussion

In this study, we leveraged genetic data to investigate the complex relationship between SDB, BMI and AF in a two-sample MR study. In univariable MR analysis, genetically predicted SDB was associated with increased risk of AF, though this relationship was lost in sensitivity analyses more robust to the presence of pleiotropic and outlying variants. Using multivariable MR analysis adjusting for genetically predicted BMI, our results suggest SDB, in general, is unlikely to be causally related to the development of AF through pathways, independent of BMI. The role of BMI in mediating the relationship between SDB and AF is highlighted by the IVW MR effect estimate becoming non-significant following inclusion of BMI in the multivariable MR model. We also show that BMI is likely to be causally related to AF, independent of SDB, even when accounting for pleiotropic or outlier SNPs. Taken together, these results suggest that BMI is a modifiable risk factor for AF while the association between SDB, in general, and AF is likely to be attributable to mediation or confounding from BMI.

### 4.1. BMI Is Causally Associated with AF

The present study further strengthens the evidence base for the causal role of obesity on AF development and confirms the results of previous MR analyses [35,36]. Obesity is a rising global health challenge that is associated with significant indirect and direct adverse cardiometabolic effects. High BMI is associated with atrial myocardial remodelling [37], atrial fibrosis [37] and epicardial fat deposition [15]. Epicardial fat is a highly pro-arrhythmic entity causing abnormal conduction and repolarisation via paracrine mechanisms and direct fatty myocardial infiltration [15]. The findings of this study, therefore, corroborate current knowledge on the strength of the association between high BMI and AF. Clinically, these results are supported by randomised data. An RCT investigating an intensive structured weight loss programme resulted in reductions in AF burden and severity [38]. In a population with persistent AF, the RACE-3 trial showed improvements in maintenance of sinus rhythm with an intervention of physical activity and dietary restrictions, alongside optimised medical therapy [39]. Altogether, our study provides further evidence to support the strength of this pathological pathway and highlights the paramount importance of risk factor modification through weight loss.

### 4.2. SDB Is not Causal for AF Independent of BMI

The results of this study did not support a causal pathway between SDB, in general, and AF, after accounting for BMI. There is a growing body of observational [8,9,10,11] and clinical trial evidence [12,13] suggesting a possible lack of direct causal association between OSA, the most common form of SDB, and AF. A recent, small RCT failed to show a benefit of CPAP against AF recurrence after cardioversion in patients with persistent AF [12], though the results should be interpreted with caution as recruitment targets were not met. Another adequately powered trial enrolling patients with paroxysmal AF failed to show a benefit of CPAP in reducing AF burden in patients with moderate to severe OSA [13]. However, there are also many studies that support an independent association between OSA and AF. Experimental models of OSA have previously shown increased vulnerability to AF [40]. An association between OSA and increased risk of AF in community and sleep clinic cohorts has been reported in observational studies [4,5,6] and observational meta-analysis reported reduced AF recurrence with CPAP therapy [7]. Similarly, a recent MR study reported a Mendelian randomisation association between genetically proxied OSA and AF, though only five SNPs were instrumented in this analysis, with two associated with BMI or whole-body, fat-free mass at the genome-wide significance level [20]. Our study builds on these data by including almost six-fold more SNPs significantly correlated with sleep apnoea traits, in addition to performing multivariable MR to control for the well-characterised, powerful pleiotropic effects of BMI on the relationship between SDB and AF.

A possible explanation to reconcile our findings with the observational studies supporting a causal link between OSA and AF is that statistical adjustment for confounders in observational studies is limited by unmeasured or unmeasurable confounding, to which MR analysis is more robust. Furthermore, multivariable regression can be limited by violation of the assumption that no included covariates are subject to collider bias or measurement imprecision [41]. Considering BMI as a covariate, if BMI measurements were subject to random error or intraindividual variation, regression coefficients would be attenuated such that the impact of BMI on the relationship between OSA and AF would be underestimated. Genetic variants strongly related with OSA and BMI generally maintain their associations throughout life, enabling the MR approach to reduce attenuation by errors and resultant bias [19]. Finally, our study looked as SDBs, in general, as the exposure, rather than OSA specifically. It is possible that the more severe forms of SDB, such as OSA, are causally associated with AF, and this effect was diluted and lost when considering all SDBs, in general. Larger future GWAS that allow for MR analyses with increased statistical power will enable further parsing of the independent effects of OSA on AF with the multivariable MR methodology.

### 4.3. Strengths and Limitations

Our study provides evidence supporting previous RCTs with the methodological strengths of the MR design, which addresses key limitations of conventional observational epidemiological studies. Principally, independent allele distribution at conception distributes confounders equally, analogous to a ‘natural’ RCT. Additionally, disease pathogenesis is unlikely to alter germline genotype, rendering associations between genotype and disease outcome less vulnerable to reverse causality [19]. This is particularly important as, following the establishment of a proarrhythmogenic atrial substrate, AF perpetuates further AF, complicating exposure–outcome interactions. Whilst clinically relevant causal effects can only be demonstrated in adequately powered and well-conducted RCTs, MR has previously predicted late-stage therapeutic failure in a phase III cardiovascular trial, where classical observational studies suggested promise [42]. Taken together with the RCTs of CPAP in AF [12,13], the present study provides further evidence to support the recently downgraded IIb recommendation of considering OSA management in patients with AF [1].

Using a GWAS for snoring as a proxy for genetically determined SDB and extrapolating to OSA is a limitation of this study. There are a few reasons why this approach was chosen. In the GWAS for snoring performed by Campos et al. [23], the trait that showed the highest genetic correlation with snoring was OSA (rG = 0.78, *p* = 3 × 10^−5^), indicating a strong association between the traits. This genetic correlation remained significant following sensitivity analysis adjusting for BMI. Similarly, significant genetic correlations were observed between snoring genes and two other measures of overnight oxyhaemoglobin saturation that are known proxies of sleep-disordered breathing: minimum SpO2 saturation, and percentage of sleep with oxyhaemoglobin saturation under 90%. Using these discovery GWAS summary statistics, Campos et al. [23] subsequently developed and validated a PGS in an independent target sample. Participants in the highest PGS decile had around twice the odds of probable OSA compared with those in the lowest decile, indicating the validity of snoring as a genetic proxy for OSA in this study.

Another limitation is that MR analysis considers associations of lifelong, cumulative genetic risk, which is not equivalent to associations measured in conventional epidemiological studies. This explains why ORs of AF per 1-SD kg/m^2^ increase in genetically predicted BMI reported in our study are larger than corresponding estimates in a large, contemporary epidemiological meta-analysis [14]. MR estimates should, therefore, not be extrapolated to infer the magnitude of effect of a clinical intervention. Additionally, summary GWAS data used in this study [22,23,24] were derived mainly from individuals of European ancestry, which may limit the generalisability of our findings to non-European ancestry populations. Although further study in these populations is warranted, using ethnically homogeneous samples in our analysis reduces the likelihood of associations between genetic variant and outcome being confounded by hidden population structure. Finally, a degree of overlap existed between the SDB and AF cohort, with a maximum estimated overlap of 78% and, therefore, a resultant risk of bias of ~7.8%. However, this would bias toward a Type 1 rather than Type 2 error and, therefore, it should not produce bias in this negative study finding.

## 5. Conclusions

In summary, our MR study explored the relationships between SDB, BMI and AF. Our results support previous observational and RCT evidence that BMI is likely to be causally related to the development of AF, while adding that these effects are likely independent of SDB. Our study suggests that an independent causal pathway between SDB, in general, and AF is unlikely. However, we cannot exclude that the more severe forms of SDB, such as OSA, are independently causally associated with AF. The RCTs currently recruiting to explore the effects of CPAP therapy on AF outcomes should further clarify the role of OSA management in patients with AF and inform clinical guidelines.

## Data Availability

All data used for this study are publicly available and their original studies are cited.

## Supplementary Materials

The following are available online at https://www.mdpi.com/article/10.3390/genes13010104/s1, Table S1: MR results investigating the effect of SDB and BMI on the primary outcome of AF, Table S2: SNP MR estimates in the analysis of SDB and AF, Table S3: SNP MR estimates in the analysis of BMI and AF.
