# Supplementary material for: Sleep Disordered Breathing, Obesity and Atrial Fibrillation: A Mendelian Randomisation Study
Source: Genes (Basel). 2022 Jan 2;13(1):104. doi: 10.3390/genes13010104 (PMC8774383; doi:10.3390/genes13010104)
Supplement: Supplementary file 1 [file genes-13-00104-s001.zip › genes-1512430-supplementary.pdf]

Supplementary Data

Sleep-disordered breathing, obesity and atrial fibrillation: a Mendelian randomisation study.

Table S1. Mendelian randomisation results investigating the effect of sleep-disordered breathing and body mass index on the primary outcome of atrial fibrillation……………………………………………………………………………………………2

Table S2. Single-nucleotide polymorphism Mendelian randomisation estimates in the analysis of sleep-disordered breathing (exposure) and atrial fibrillation (outcome)………………..……………………………………………………………………3

Table S3. Single-nucleotide polymorphism Mendelian randomisation estimates in the analysis of body mass index (exposure) and atrial fibrillation (outcome)…………… ……………………………………………………………..........................5

Table S1 – Mendelian randomisation results investigating the effect of sleep-disordered breathing (SDB) and body mass index (BMI) on the primary outcome of atrial fibrillation.

**Exposure**	**Method**	**Number of SNPs**	**p-value**	**OR**	**LCI 95%**	**UCI 95%**
SDB (↑ logOR snoring)	IVW-MRE	29	0.03	2.09	1.10	3.98
	Weighted median		0.32	1.40	0.72	2.70
	MR Egger*		0.40	0.24	0.01	6.53
	MR-PRESSO†		0.06	1.85	1.00	3.41
	Multivariable MR‡		0.12	0.68	0.42	1.10
BMI (↑ 1-SD kg/m^2)	IVW-MRE	453	<0.001	1.33	1.24	1.42
	Weighted median		<0.001	1.36	1.26	1.47
	MR Egger§		<0.001	1.52	1.26	1.83
	MR-PRESSO‖		<0.001	1.34	1.26	1.42
	Multivariable MR#		<0.001	1.40	1.30	1.51

*MR-Egger intercept 0.02, standard error 0.01, p=0.20

†n=3 outlier SNPs excluded, global MR-PRESSO p<0.001

‡Adjusted for genetically-predicted BMI

§MR-Egger intercept -2x10^{-3}, standard error 1x10^{-3}, p=0.14

‖n= 10 outlier SNPs excluded, global MR-PRESSO p<0.001

#Adjusted for genetically-predicted SDB

IVW-MRE = inverse variance weighted with mixed random effects, MR = mendelian randomisation, SNP = single-nucleotide polymorphism, LCI= lower confidence interval, UCI = upper confidence interval

Table S2 – Single-nucleotide polymorphism (SNP) Mendelian randomisation estimates in the analysis of sleep-disordered breathing (SDB, exposure) and atrial fibrillation (AF, outcome). Beta = beta coefficients, which represent the genetic association estimate produced by dividing genetic association of the SNP with the outcome (AF, units of log odds ratio atrial fibrillation liability) by genetic association of the SNP with the exposure (SDB, units of log odds ratio snoring liability).

SNP	Chromosome	Position	Beta	Standard error	p-value
rs10878269	12	65791463	-1.772723321	0.835551119	0.033869525
rs11075985	16	53805207	3.200824973	1.042802706	0.002144573
rs1108431	16	31054607	1.698578199	1.137440758	0.135349954
rs12119849	1	96878072	0.481389011	1.085165059	0.657325281
rs12429765	13	40745860	0.529449138	1.058898275	0.617075077
rs13156484	5	122653399	2.760495923	1.242223165	0.026268291
rs13251292	8	71474355	-0.528833637	1.016987764	0.603063575
rs1374895	3	77615539	-0.725603138	1.09612389	0.507989275
rs1609721	3	94009049	-0.973460818	1.204451521	0.418964419
rs1641511	17	7559677	4.604090223	1.147524007	6.02E-05
rs17060460	6	100827834	-0.789693373	1.170438393	0.49986715
rs17413833	2	103900735	2.986745729	1.313542624	0.022977441
rs17680229	17	46129762	1.057493498	1.178664628	0.3696142

rs180110	17	67930613	-0.808652285	1.073302124	0.45119477
rs2207944	6	84307328	0.235480919	1.211044728	0.845827892
rs2307111	5	75003678	2.621478122	0.939036939	0.005243734
rs2664299	14	99742187	0.506468131	1.012936262	0.617075077
rs34811474	4	25408838	3.138963542	1.18805393	0.008239244
rs4976269	5	134452597	-0.818244515	1.110474699	0.461218375
rs4987719	18	60960310	-0.382131059	1.590158276	0.810089883
rs57222984	17	43758898	4.114045613	1.07889957	1.37186E-04
rs592333	13	51340315	0.529921814	0.816962797	0.516565503
rs6054427	20	6635266	2.754707542	1.234868898	0.025696419
rs6099273	20	55347828	0.299313375	1.272081844	0.81398046
rs61597598	2	156996626	0.042035528	0.91637452	0.963412622
rs725861	10	9063776	-0.121118963	0.990973334	0.902723028
rs8069947	17	1985843	0.136240194	1.089921556	0.90052355
rs8108822	19	32183171	-2.124548189	1.066872683	0.046439202
rs947612	6	73738661	1.902242566	1.218624144	0.118529506

Table S3 – Single-nucleotide polymorphism (SNP) Mendelian randomisation estimates in the analysis of body mass index (BMI, exposure) and atrial fibrillation (AF, outcome). Beta = beta coefficients, which represent the genetic association estimate produced by dividing genetic association of the SNP with the outcome (AF, units of log odds ratio atrial fibrillation liability) by genetic association of the SNP with the exposure (BMI, units of 1-SD kg/m^2).

SNP	Chromosome	Position	Beta	SE	p-value
rs10002111	4	67815504	0.576	0.704	0.413253375
rs10033843	4	77028783	-0.235714286	0.614285714	0.701185298
rs10050620	5	63927239	-0.661538462	0.584615385	0.257811495
rs1006317	1	209552636	0.2625	0.65625	0.689156517
rs10099330	8	143383694	0.991596639	0.596638655	0.096518423
rs10101364	8	20634888	0.316666667	0.658333333	0.630507502
rs10110189	8	15393380	-1.448717949	0.756410256	0.055460089
rs10132280	14	25928179	0.570093458	0.373831776	0.127259098
rs10145749	14	102782109	-0.683229814	0.608695652	0.261671589
rs10168563	2	35552173	0.912698413	0.626984127	0.14547662
rs10169594	2	41637688	1.65	0.641666667	0.010127991
rs10182181	2	25150296	-0.29969419	0.217125382	0.16749993
rs10185199	2	40282202	0.083916084	0.573426573	0.883651835
rs10197031	2	105454590	0.279503106	0.48447205	0.563991421

rs10261050	7	114337652	-0.371681416	0.637168142	0.559668927
rs1048637	3	13358171	0.234042553	0.755319149	0.756668065
rs10499694	7	50614173	0.5	0.546153846	0.359932898
rs10506971	12	89757937	0.563380282	0.5	0.259843728
rs10510419	3	12426936	0.785714286	0.625	0.208701911
rs10518694	15	53072673	0.215277778	0.701388889	0.758896126
rs10733051	1	167280354	-1.333333333	0.774193548	0.085029271
rs10733682	9	129460914	0.398648649	0.486486486	0.412532886
rs10741329	11	89997796	-0.2	0.67826087	0.768091819
rs10742752	11	45438374	-0.414634146	0.593495935	0.484783205
rs1075901	17	15943910	0.889830508	0.618644068	0.150333024
rs10761785	10	65318766	-4.496240602	0.639097744	1.99E-12
rs10772983	12	17141582	-0.28	0.71	0.693310697
rs10779751	1	11284336	0.488549618	0.633587786	0.440656944
rs10811868	9	23199959	0.73	0.77	0.343103009
rs10829164	10	27318370	1.145695364	0.635761589	0.071532295
rs10864728	1	230304914	-0.236363636	0.663636364	0.721717463
rs10909880	1	2727804	0.251851852	0.540740741	0.641392007

rs10920336	1	202115945	-0.861386139	0.722772277	0.233347198
rs10920678	1	190239907	0.295302013	0.476510067	0.535443259
rs10929925	2	6155557	0.816901408	0.5	0.102300261
rs10938397	4	45182527	0.49378882	0.223602484	0.027221045
rs10942267	5	80841914	-0.168918919	0.527027027	0.748579613
rs10961649	9	14670949	0.923076923	0.740384615	0.212487988
rs10968114	9	27800007	-0.008849558	0.646017699	0.989070416
rs10992867	9	96461013	0.382716049	0.49382716	0.43833966
rs11030618	11	29243293	-0.236363636	0.654545455	0.718016387
rs11046972	12	23705969	0.693181818	0.698863636	0.321260985
rs11060853	12	123424071	-0.523364486	0.700934579	0.455264795
rs11066188	12	112610714	-1.026315789	0.675438596	0.128641588
rs11115176	12	82465797	-0.72519084	0.648854962	0.263717762
rs11121210	1	8708529	1.342342342	0.675675676	0.046959348
rs11128021	3	88139016	-0.337016575	0.569060773	0.553694568
rs11150911	18	73498528	0.059322034	0.669491525	0.929393772
rs11165643	1	96924097	0.508108108	0.394594595	0.197860431
rs11170468	12	39430048	-0.4	0.669230769	0.550039358

rs11218510	11	121922587	-0.128571429	0.528571429	0.807816969
rs11246136	11	371265	-0.263473054	0.814371257	0.746294337
rs112646560	1	39560250	0.605555556	0.488888889	0.215480195
rs11525873	7	138817193	0.965517241	0.487068966	0.047445575
rs11538	22	18220831	1.623188406	0.717391304	0.023658738
rs11577094	1	38026600	0.602150538	0.741935484	0.417024519
rs11594179	10	104392580	0.100917431	0.816513761	0.901635551
rs1159692	5	63977815	-0.555555556	0.555555556	0.317310508
rs11611246	12	939480	0.098654709	0.394618834	0.802587349
rs11614340	12	133426483	0.982905983	0.666666667	0.140384964
rs11615578	12	121714935	0.547008547	0.743589744	0.461954571
rs11633626	15	95271378	-0.057324841	0.47133758	0.903198689
rs11636611	15	36391965	0.519230769	0.692307692	0.453254705
rs116374395	5	50723410	0.657320872	0.619937695	0.289007454
rs11649864	17	56093061	1.276041667	0.677083333	0.059481784
rs11655587	17	47140794	-0.271428571	0.361904762	0.453254705
rs11672660	19	46180184	-0.402366864	0.26035503	0.122236355
rs1168	2	191829777	-0.42295082	0.239344262	0.077207583

rs11692326	2	208263279	1.659863946	0.578231293	0.004097088
rs11695013	2	157057487	-0.432692308	0.721153846	0.548506236
rs11713193	3	49924424	0.170731707	0.292682927	0.559668927
rs11739877	5	105876806	0.603448276	0.629310345	0.337607054
rs11757278	6	13180454	0.135338346	0.571428571	0.812779285
rs11772246	7	71603692	0.344827586	0.627586207	0.582696292
rs11773362	7	147668180	0.076190476	0.714285714	0.915053428
rs11782074	8	142617096	-0.290322581	0.612903226	0.635725115
rs118081010	11	46174948	0.451737452	0.629343629	0.47288591
rs11882409	19	34019685	0.644628099	0.669421488	0.33556611
rs11902450	2	12845368	0.658682635	0.724550898	0.363302141
rs11915371	3	70539559	0.136363636	0.584415584	0.815502577
rs11921432	3	35117776	0.920634921	0.634920635	0.147058519
rs12072739	1	98315893	-0.076923077	0.514792899	0.881218059
rs12098284	10	76047464	1.293478261	0.581521739	0.02612833
rs12140153	1	62579891	-0.274787535	0.382436261	0.472437634
rs12150665	17	34914787	-0.011904762	0.428571429	0.97783939
rs12259464	10	53680099	-0.376146789	0.678899083	0.579541853

rs12282785	11	76476030	0.044585987	0.547770701	0.935127548
rs12286929	11	115022404	-0.378531073	0.406779661	0.352083514
rs12334877	8	67194171	-0.423611111	0.611111111	0.488195457
rs12364470	11	134601012	0.802139037	0.540106952	0.137504487
rs12369179	12	122963550	0.7	0.402941176	0.082347238
rs12386885	8	87766769	0.602649007	0.668874172	0.367593584
rs12421848	11	891338	-0.262411348	0.524822695	0.617075077
rs12429545	13	54102206	0.463258786	0.332268371	0.163247929
rs12448257	16	3599655	1.428571429	0.540372671	0.008201059
rs12462975	19	30272202	0.25388601	0.404145078	0.529869571
rs12527426	6	153392002	-0.46	0.513333333	0.370197276
rs12591120	15	99236869	0.741666667	0.691666667	0.283590176
rs12602912	17	65870073	0.180722892	0.524096386	0.730223993
rs12611148	19	19865077	0.131386861	0.715328467	0.854269623
rs12628051	22	40654276	0.149068323	0.459627329	0.745692506
rs12628891	22	38317137	-0.443478261	0.67826087	0.513210928
rs12636480	3	82719412	0.2421875	0.5859375	0.679362411
rs12652212	5	88808594	0.297709924	0.549618321	0.588048157

rs1268065	6	126042783	1.66	0.72	0.021135479
rs12680842	8	95582606	0.464788732	0.528169014	0.37885931
rs12681792	8	62054463	-0.54	0.593333333	0.362763257
rs12692596	2	161265910	0.808333333	0.616666667	0.189921689
rs12714199	2	86812549	2.120567376	0.517730496	4.21E-05
rs12765914	10	34013507	-0.712389381	0.548672566	0.194154351
rs12888545	14	88308044	-0.315789474	0.654135338	0.629267135
rs12912198	15	47103953	0.94	0.82	0.251653893
rs12926250	16	72213316	-0.427777778	0.672222222	0.524539436
rs1293037	6	70248345	0.713178295	0.651162791	0.27341234
rs12939549	17	78611724	0.244444444	0.394444444	0.535443259
rs1296328	4	137083193	0.885542169	0.451807229	0.04999579
rs12981256	19	1865901	1.039735099	0.496688742	0.036319409
rs13021737	2	632348	0.477508651	0.166089965	0.004040275
rs13033310	2	133523605	-0.020547945	0.554794521	0.970455475
rs1304549	20	54378256	0.466101695	0.754237288	0.536590152
rs13107325	4	103188709	0.006410256	0.318376068	0.983936296
rs13110266	4	162129844	0.411290323	0.580645161	0.478738282

rs13174863	5	139080745	-0.116751269	0.527918782	0.824972645
rs13186194	5	60795485	2.02	0.75	0.00707415
rs13191362	6	163033350	0.919148936	0.493617021	0.06259337
rs13240600	7	99064466	-0.366666667	0.527777778	0.487220235
rs13245051	7	113362799	0.993333333	0.506666667	0.049934305
rs13263601	8	14095900	0.875862069	0.524137931	0.094711282
rs1327259	6	51177811	-0.050955414	0.47133758	0.913909937
rs13296413	9	37258105	0.533783784	0.5	0.28571562
rs13298487	9	126112104	0.782608696	0.695652174	0.260589034
rs1346841	4	65651730	-0.03968254	0.579365079	0.945393072
rs1350430	12	41819215	0.088	0.568	0.876876836
rs1356506	18	40708038	0.299270073	0.532846715	0.574358708
rs1358980	6	43764551	1.496124031	0.558139535	0.007350006
rs1383592	8	106430676	-0.803278689	0.713114754	0.259980685
rs1409818	20	21381121	-0.394871795	0.594871795	0.50682274
rs1421334	8	30865733	0.259259259	0.540740741	0.631617069
rs1437842	4	173597016	0.452830189	0.669811321	0.499004876
rs1441264	13	79580919	0.287356322	0.41954023	0.493387143

rs1451077	2	147901207	0.278106509	0.426035503	0.513899552
rs147568678	10	93061851	0.589552239	0.656716418	0.369330966
rs1477199	16	53712135	0.199095023	0.470588235	0.672239139
rs1492014	3	94071481	-0.321637427	0.421052632	0.444933459
rs1492767	4	55221467	0.315789474	0.757894737	0.676922239
rs1501673	5	87963600	0.242214533	0.335640138	0.470510007
rs1522569	4	171632637	-0.219858156	0.680851064	0.746758354
rs1559673	15	62156514	1.200557103	0.509749304	0.018513174
rs16851483	3	141275436	0.647727273	0.375	0.084118695
rs16906838	8	138213836	0.137651822	0.704453441	0.845078017
rs17020497	2	81826131	-0.242857143	0.75	0.746082245
rs17024393	1	110154688	0.254658385	0.343167702	0.458037989
rs17066856	18	58049656	0.285714286	0.34	0.400719947
rs17094222	10	102395440	1.028901734	0.502890173	0.040758632
rs17149254	7	76634463	0.928571429	0.474789916	0.050494351
rs17182027	14	73348130	-1.081818182	0.663636364	0.103072548
rs17207196	7	75101065	1.190909091	0.359090909	0.00091167
rs1721447	7	109214139	-0.16	0.71	0.821705362

rs17367750	4	140782542	0.049180328	0.631147541	0.937890037
rs17405819	8	76806584	0.440758294	0.36492891	0.227127199
rs17544384	1	115295160	0.221374046	0.702290076	0.752596602
rs17636031	10	126594078	1.266233766	0.564935065	0.025001516
rs17681451	3	114399296	0.36	0.613333333	0.557232908
rs17724992	19	18454825	1.180232558	0.470930233	0.012204594
rs17783165	18	63461638	1.375	0.6015625	0.022270979
rs17806224	20	51065854	0.561538462	0.365384615	0.12433198
rs17814208	2	144037998	0.643410853	0.651162791	0.323106008
rs1799923	3	42306294	-0.598214286	0.464285714	0.197585349
rs1808629	8	73435964	0.326732673	0.381188119	0.391365938
rs185350	19	34306816	1.04379562	0.532846715	0.050124018
rs1860561	12	110783241	-1.176100629	0.566037736	0.037729832
rs1877875	9	120664469	-0.724770642	0.660550459	0.272544261
rs1884389	20	1410582	-0.638888889	0.666666667	0.3378947
rs1884897	20	6612832	0.934782609	0.413043478	0.023625958
rs1927790	13	96922191	-0.014285714	0.514285714	0.97783939
rs1928295	9	120378483	1.126865672	0.529850746	0.033439983

rs1941213	11	133125329	-0.166666667	0.740740741	0.821979274
rs1941696	18	31252129	0.214285714	0.642857143	0.73888268
rs1945160	18	22164216	-0.019230769	0.701923077	0.978142883
rs1948080	9	11852043	0.455882353	0.558823529	0.414620555
rs194809	16	23804956	-0.015873016	0.714285714	0.982270691
rs1951455	14	91512339	0.898648649	0.547297297	0.100595121
rs1965529	7	77825707	0.459627329	0.540372671	0.395005641
rs197374	1	112289983	0.106382979	0.517730496	0.83719755
rs1999433	9	81371441	0.504672897	0.663551402	0.446917899
rs2007518	11	132639606	0.146153846	0.569230769	0.797366554
rs2051559	4	3298800	-0.113772455	0.622754491	0.855039452
rs2053682	5	170599327	0.447058824	0.458823529	0.329878332
rs2058527	16	6704749	0.669565217	0.695652174	0.335798503
rs2064044	21	22119890	-0.008130081	0.739837398	0.991232214
rs2065418	11	30422068	2.618705036	0.532374101	8.70E-07
rs2066295	6	26168903	0.338028169	0.598591549	0.572273833
rs2074314	11	17411821	0.276190476	0.704761905	0.695138094
rs2108719	7	39481056	0.308411215	0.747663551	0.679972981

rs2112347	5	75015242	0.706521739	0.268115942	0.008410379
rs2119753	2	151224579	0.21	0.73	0.773598412
rs2120710	8	93210803	0.712871287	0.752475248	0.343451077
rs2134858	9	73837155	0.982905983	0.606837607	0.105292801
rs213518	7	26941065	0.431372549	0.692810458	0.533520127
rs214249	16	348687	0.007246377	0.536231884	0.989218104
rs215669	7	32378979	0.241610738	0.489932886	0.62190612
rs217433	7	44553496	1.034782609	0.8	0.195845698
rs217669	14	62360075	-0.162790698	0.459302326	0.723016371
rs2192158	4	55505360	0.481751825	0.518248175	0.3525899
rs2196618	8	85089437	-0.01459854	0.591240876	0.980301148
rs2206277	6	50798526	0.399509804	0.218137255	0.067031812
rs2228213	6	12124855	1.083333333	0.520833333	0.037525533
rs2228552	1	32165495	1.322580645	0.596774194	0.026676698
rs2238799	22	20109325	1.039215686	0.715686275	0.14648637
rs2241423	15	68086838	0.33557047	0.27852349	0.228273076
rs2246012	6	131898208	0.956521739	0.571428571	0.09414769
rs2257791	10	118643670	0.338129496	0.589928058	0.566529099

rs225882	14	30480123	1.477876106	0.699115044	0.034521755
rs2267958	9	131015279	0.538461538	0.584615385	0.357022949
rs2271189	12	56494991	-0.496453901	0.517730496	0.337607054
rs2273175	14	104160141	-1.107438017	0.628099174	0.077873863
rs2275003	9	34124860	-1	0.63963964	0.117963175
rs2283006	7	93085722	0.560606061	0.553030303	0.310726578
rs2283093	7	126721231	0.016528926	0.727272727	0.981867821
rs2289379	7	44804225	1.328467153	0.547445255	0.015238249
rs2342892	16	24540806	-0.952380952	0.571428571	0.095580705
rs2357760	6	120213880	1.167832168	0.524475524	0.02596956
rs2365389	3	61236462	1.011904762	0.44047619	0.021601816
rs2400414	1	194965200	-0.325396825	0.587301587	0.579541853
rs2423668	20	12430673	0.29245283	0.698113208	0.67527539
rs2436728	6	40365601	0.64021164	0.375661376	0.088338995
rs2439823	10	99778226	-0.024242424	0.436363636	0.955695871
rs2466103	8	32412304	-1.743801653	0.669421488	0.009189081
rs2470893	15	75019449	-0.130841121	0.757009346	0.862777583
rs2503185	1	66461401	1.076923077	0.553846154	0.051841879

rs2513999	11	103019633	-0.344155844	0.649350649	0.596111931
rs2600226	3	12928762	-1.612068966	0.646551724	0.012654998
rs2605603	11	93221105	1.708737864	0.699029126	0.014507542
rs2622274	6	64240516	0.757009346	0.663551402	0.253934401
rs264941	2	104297420	1.64516129	0.572580645	0.004062861
rs2707183	12	116957607	0.376344086	0.76344086	0.622042443
rs2712665	12	99594947	-0.175925926	0.75	0.814544126
rs2715423	15	99511873	0.773913043	0.72173913	0.283590176
rs273512	19	18224729	0.794871795	0.467948718	0.089388894
rs2744974	6	34579431	-0.639846743	0.287356322	0.02596956
rs274628	7	86265855	-0.294117647	0.745098039	0.69303711
rs2777768	9	84186734	1.680672269	0.705882353	0.017267944
rs2820295	1	201800868	0.659574468	0.327659574	0.044115996
rs2832283	21	30690558	0.208695652	0.782608696	0.789725821
rs28350	3	42418446	0.75	0.552325581	0.174497088
rs28489620	22	41804716	0.920529801	0.562913907	0.101987406
rs2861685	2	67837553	1.024242424	0.436363636	0.01891397
rs2862996	11	43653833	0.430555556	0.361111111	0.233140619

rs2907948	7	150638484	-3.434482759	0.593103448	7.01E-09
rs2910026	5	152529936	-0.189393939	0.621212121	0.760459035
rs2962334	5	86879056	1.045454545	0.641414141	0.10311814
rs2984618	1	47690438	-1.012121212	0.460606061	0.027994145
rs3019466	11	92476178	0.5078125	0.7734375	0.511460238
rs305256	8	137568252	0.026086957	0.739130435	0.971845214
rs3101336	1	72751185	0.480314961	0.295275591	0.103807912
rs3115667	6	31643399	-0.055865922	0.474860335	0.906347316
rs312750	17	68343539	4.329896907	0.731958763	3.31E-09
rs321237	1	96478125	0.30952381	0.666666667	0.642443058
rs326893	4	112691776	1.842975207	0.603305785	0.002252148
rs329651	11	133767622	-0.0625	0.6	0.917037086
rs337637	4	38604470	0.306569343	0.540145985	0.570328635
rs339991	15	60913637	0.688	0.568	0.22579286
rs34184235	3	86192846	-0.026086957	0.626086957	0.966764427
rs34234296	2	175166636	1.048275862	0.524137931	0.045500264
rs34517439	1	78450517	0.749360614	0.309462916	0.015457128
rs34811474	4	25408838	0.85665529	0.324232082	0.008239244

rs349088	11	84814393	-0.176923077	0.546153846	0.745980687
rs35408866	4	187743245	-1.371069182	0.72327044	0.058006053
rs35483388	11	122545146	0.192	0.584	0.742331715
rs35867081	17	79047278	0.22	0.493333333	0.655636292
rs35949039	16	70572605	0.948837209	0.553488372	0.086476265
rs3732927	3	170586057	0.191919192	0.757575758	0.80001064
rs3764625	19	49649051	-0.145833333	0.770833333	0.84994454
rs3764835	2	159519368	-0.715384615	0.769230769	0.352371084
rs3770890	2	36657992	2.531986532	0.808080808	0.00172833
rs3796432	4	96030402	0.03539823	0.663716814	0.957466322
rs3806114	6	20482335	-0.983333333	0.65	0.130325257
rs3808477	8	116670347	1.28021978	0.43956044	0.003585482
rs3814883	16	29994922	0.629955947	0.321585903	0.050124018
rs3825061	11	118944675	1.3	0.528571429	0.013914641
rs3902951	14	69789755	0.358208955	0.619402985	0.563052648
rs3914628	4	147438019	1.690909091	0.606060606	0.005270804
rs3923783	17	1843189	0.59009009	0.423423423	0.163433286
rs39654	3	173095123	0.779141104	0.441717791	0.077750688

rs40067	5	107439012	-0.182539683	0.361111111	0.613211557
rs4017425	3	44028764	-0.830508475	0.601694915	0.16749993
rs4097319	10	33860515	1	0.672897196	0.137249734
rs4148155	4	89054667	0.869791667	0.567708333	0.12549527
rs4240673	8	10787612	-0.651428571	0.422857143	0.123428649
rs427943	21	46570896	0.457627119	0.406779661	0.260589034
rs4286488	4	94440026	-0.32231405	0.694214876	0.642443058
rs429358	19	45411941	0.194552529	0.40077821	0.627366467
rs4303732	2	100830040	-0.159763314	0.431952663	0.711484561
rs4307239	7	24354300	-0.208695652	0.634782609	0.742331715
rs4390583	16	81694835	-1.36	0.74	0.066086304
rs4430672	14	63094407	-0.080645161	0.701612903	0.908490675
rs4482463	2	205375909	0.461290323	0.383870968	0.229487272
rs45486197	19	2244849	-0.666666667	0.602150538	0.268232169
rs4653017	1	33776728	0.974576271	0.661016949	0.140384964
rs4655141	1	23312025	-0.473988439	0.537572254	0.37792801
rs4671328	2	58935282	-0.476635514	0.336448598	0.156580407
rs4700608	5	63026280	-0.425806452	0.464516129	0.359317338

rs4721089	7	1872921	0.676646707	0.526946108	0.199110149
rs4740383	9	133783566	0.549618321	0.557251908	0.323985248
rs4740619	9	15634326	0.507936508	0.380952381	0.182422439
rs474605	18	39612720	-0.072	0.592	0.903198689
rs4771218	13	28655311	0.241134752	0.539007092	0.654609073
rs478707	18	7543207	-0.806666667	0.586666667	0.169131445
rs4812405	20	35276585	0.851485149	0.772277228	0.270216507
rs4813619	20	2815715	-0.990566038	0.698113208	0.155922656
rs4858193	3	20441050	0.92481203	0.62406015	0.138359531
rs4864201	4	130731284	0.839416058	0.547445255	0.125193746
rs4865796	5	53272664	1.5625	0.8125	0.05447039
rs4880341	10	133992689	1.015384615	0.576923077	0.078407807
rs4900714	14	47302219	0.013333333	0.48	0.97783939
rs4921301	5	159984492	0.534883721	0.674418605	0.427717554
rs4970991	1	151004003	-1.163793103	0.75862069	0.125007289
rs4973618	2	229002620	0.695945946	0.493243243	0.158256732
rs4981693	14	29680331	-0.514851485	0.410891089	0.210201322
rs4986044	17	21261560	0.276836158	0.406779661	0.496152757

rs4988235	2	136608646	1.129032258	0.701612903	0.107573621
rs543874	1	177889480	0.244258873	0.183716075	0.18366808
rs56133507	2	172818467	1.580152672	0.72519084	0.029335578
rs56151256	15	78024806	0.397590361	0.530120482	0.453254705
rs56211164	7	158016764	-0.8984375	0.6796875	0.186221742
rs562664	11	63823619	0.471014493	0.695652174	0.498353083
rs570463	11	28739318	0.661157025	0.661157025	0.317310508
rs57989773	6	100629078	2.507042254	0.64084507	9.15E-05
rs587271	1	54743111	0.891666667	0.708333333	0.20809408
rs592483	11	69445173	1.094890511	0.532846715	0.039898856
rs6010784	20	61540319	-0.698113208	0.679245283	0.304054372
rs61740466	1	19934900	-0.35915493	0.584507042	0.53891283
rs61813324	1	156049877	0.103806228	0.401384083	0.795927918
rs61828641	1	174321997	0.874439462	0.497757848	0.078959279
rs61983990	14	41475003	0.515151515	0.742424242	0.487759003
rs6265	11	27679916	-0.072639225	0.215496368	0.73605761
rs6443750	3	181329682	-0.322368421	0.657894737	0.624133899
rs6445258	3	62112198	-0.572519084	0.709923664	0.419982497

rs6463489	7	5542513	1.167664671	0.71257485	0.101285035
rs6470144	8	124152245	-1.367346939	0.755102041	0.070170142
rs6493498	15	51754451	0.153284672	0.518248175	0.767402185
rs6500208	16	49011249	1.561643836	0.589041096	0.008021517
rs650198	12	69674595	0.00729927	0.576642336	0.989900465
rs6545714	2	59307725	0.257731959	0.371134021	0.48740353
rs6556301	5	176527577	-0.973451327	0.654867257	0.137150489
rs6567160	18	57829135	0.445652174	0.152173913	0.003405236
rs657452	1	49589847	0.468085106	0.388297872	0.228018266
rs6591407	11	56914157	0.403225806	0.741935484	0.586800555
rs6607337	17	35057373	0.161290323	0.629032258	0.797634069
rs6656785	1	75005776	0.308988764	0.415730337	0.457334427
rs66595146	18	58204315	1.488888889	0.548148148	0.006603278
rs6661316	1	210095527	-0.766666667	0.6	0.201327792
rs6720868	2	230663576	0.162337662	0.493506494	0.74219547
rs6725931	2	220205146	0.556149733	0.545454545	0.307914484
rs6783054	3	11672805	-2.474747475	0.717171717	5.59126E-04
rs6803161	3	196205694	0.242990654	0.691588785	0.725324775

rs6804842	3	25106437	1.411347518	0.510638298	0.005711699
rs6808814	3	116852469	-0.016949153	0.686440678	0.980301148
rs6850421	4	180187034	0.911504425	0.637168142	0.152557633
rs6864049	5	124330522	0.909090909	0.595041322	0.126567721
rs687339	3	135932359	1.45212766	0.45212766	0.001319224
rs6882366	5	95864693	0.396946565	0.549618321	0.470157863
rs6886072	5	136598460	-0.49	0.71	0.490105619
rs6888194	5	106910657	-0.385826772	0.787401575	0.624133899
rs6890310	5	27193573	0.243697479	0.680672269	0.720324836
rs6893539	5	122705737	0.852459016	0.631147541	0.176807784
rs6909685	6	97753952	-0.067114094	0.510067114	0.895317338
rs6915002	6	54028069	-0.858585859	0.737373737	0.244268606
rs6921533	6	73742334	0.961538462	0.759615385	0.205576536
rs6922607	6	142703483	-0.630769231	0.7	0.367535737
rs6950388	7	1270699	-0.37037037	0.674074074	0.582696292
rs6973656	7	77422583	2.138613861	0.722772277	0.003087352
rs698147	5	3513485	-0.060344828	0.612068966	0.92146258
rs7024334	9	109072075	-0.659259259	0.622222222	0.289361292

rs7070670	10	61842645	-0.19047619	0.626984127	0.761282196
rs7084454	10	21821274	0.015151515	0.398989899	0.969707867
rs7102454	11	65594820	0.482142857	0.44047619	0.273694285
rs7124681	11	47529947	0.245136187	0.284046693	0.388129935
rs7138803	12	50247468	0.713804714	0.245791246	0.003682992
rs7144011	14	79940383	1.266159696	0.338403042	1.82872E-04
rs7161194	14	101529005	0.826315789	0.442105263	0.06161619
rs7171864	15	73227249	-1.098484848	0.568181818	0.053195148
rs7172627	15	31877690	0.771929825	0.649122807	0.234365228
rs7245985	19	30710410	-0.075630252	0.756302521	0.920344325
rs72649373	4	80609966	-0.113772455	0.652694611	0.861620327
rs72673947	8	118884379	-0.035874439	0.506726457	0.943559749
rs72757415	15	92572762	0.503144654	0.572327044	0.379335738
rs73225274	8	21088909	-0.461538462	0.705128205	0.512760453
rs7357754	9	92207308	-0.566666667	0.6	0.344942579
rs73985439	2	212299249	0.458015267	0.58778626	0.435849672
rs742748	20	39293397	-0.796460177	0.646017699	0.217621765
rs74887628	1	147032779	0.409836066	0.744262295	0.581866226

rs7498665	16	28883241	0.315789474	0.259649123	0.223902568
rs750090	4	152931436	-1.265486726	0.663716814	0.056563766
rs7512146	1	34283008	0.329896907	0.731958763	0.652202737
rs7534091	1	118864616	0.283333333	0.666666667	0.670836675
rs7561278	2	48954905	0.431952663	0.514792899	0.40142416
rs756717	16	72996162	-1.753731343	0.544776119	0.001285586
rs7588437	2	181575281	0.781818182	0.454545455	0.085432442
rs7593917	2	203931012	0.869565217	0.626086957	0.16486654
rs7599312	2	213413231	0.565934066	0.456043956	0.214619102
rs7616009	3	194881756	-0.78343949	0.617834395	0.204783225
rs7631156	3	131751628	0.362790698	0.362790698	0.317310508
rs7640424	3	107820063	2.111111111	0.607407407	5.09693E-04
rs765125	12	2156207	0.214285714	0.755102041	0.776576083
rs765875	6	143185683	0.492424242	0.537878788	0.359932898
rs76638898	10	21099584	0.720744681	0.752659574	0.338265925
rs7678054	4	95093855	-2.575757576	0.717171717	3.28718E-04
rs768023	6	108876002	-1.055900621	0.453416149	0.019871406
rs76942203	11	116973247	1.593155894	0.562737643	0.004639096

rs7696649	4	120322177	1.043103448	0.715517241	0.144887042
rs7713317	5	95716722	0.909638554	0.469879518	0.052880264
rs7715256	5	153537893	0.683544304	0.46835443	0.144438691
rs77165542	2	430975	0.52715655	0.260915868	0.043341067
rs7727781	5	165185571	0.150537634	0.774193548	0.845827892
rs7730004	5	43191033	1.158273381	0.54676259	0.034139424
rs7734385	5	158460212	-0.732673267	0.712871287	0.304054372
rs77432547	13	86494817	0.441176471	0.482352941	0.360383726
rs7760482	6	147354276	-1.156862745	0.715686275	0.105999571
rs7774	17	4801163	-0.265625	0.609375	0.662911146
rs7802342	7	137435925	0.209677419	0.637096774	0.74206958
rs7842934	8	132838921	0.382022472	0.696629213	0.583426134
rs7861160	9	80799579	-0.042105263	0.768421053	0.956302166
rs7893571	10	16750129	-1.072	0.624	0.08580597
rs7899106	10	87410904	0.596330275	0.525993884	0.256911627
rs7903146	10	114758349	0.93258427	0.460674157	0.042930015
rs7907470	10	10268989	0.062146893	0.734463277	0.932567168
rs79113395	1	1590521	0.245	0.545	0.653041233

rs79186842	20	47689036	0.227722772	0.53960396	0.673011625
rs7944782	11	130795698	0.722222222	0.493055556	0.142978567
rs7975187	12	60964108	-0.299270073	0.620437956	0.629555245
rs79780963	10	104952499	1.635245902	0.508196721	0.00129204
rs79906980	1	57887985	0.310126582	0.664556962	0.640738382
rs805412	2	54120820	-2.081632653	0.734693878	0.004606532
rs8057911	16	54143352	-0.056	0.696	0.935871591
rs8065172	17	31456969	-0.120967742	0.669354839	0.85658509
rs8122855	20	25192049	0.226277372	0.554744526	0.683350957
rs8126575	21	46435610	0.427631579	0.677631579	0.527996089
rs8134638	21	40644170	-1.977443609	0.563909774	4.53757E-04
rs8181823	13	65477940	-0.92	0.672	0.17098439
rs845084	10	125220036	0.551470588	0.580882353	0.342433943
rs852056	20	17102860	0.975609756	0.682926829	0.153127451
rs865809	3	183997735	2.943548387	0.669354839	1.09E-05
rs879620	16	4015729	0.283185841	0.327433628	0.387112964
rs889398	16	69556715	0.220512821	0.379487179	0.561185809
rs891387	18	21103909	1.399038462	0.350961538	6.71E-05

rs900144	11	13294268	0.871621622	0.493243243	0.077207583
rs9168	13	99101583	-0.582733813	0.575539568	0.311299047
rs9294260	6	83433228	-0.242857143	0.521428571	0.641392007
rs9299	17	46669430	0.705882353	0.621848739	0.256318702
rs930295	2	50233352	-0.115384615	0.471153846	0.806535442
rs9320823	6	98429337	0.066666667	0.436363636	0.878573523
rs935166	2	26949366	-0.671052632	0.473684211	0.156580407
rs9370410	6	55171842	1.057142857	0.761904762	0.165289351
rs9375702	6	130384187	2.009433962	0.726415094	0.005670787
rs942066	14	94031914	0.554455446	0.376237624	0.140566636
rs9458814	6	163771305	-0.517857143	0.741071429	0.484680041
rs946824	1	243684019	-1.274111675	0.54822335	0.020121524
rs9478496	6	154333183	0.242038217	0.624203822	0.698197274
rs9512648	13	27933910	-1.126315789	0.757894737	0.137249734
rs9522183	13	111977280	-0.333333333	0.562962963	0.553780103
rs9527895	13	59367767	1.810126582	0.620253165	0.003518696
rs9569777	13	58484786	-0.467661692	0.452736318	0.301619327
rs9595908	13	33184288	1.051948052	0.487012987	0.03077267

rs9599161	13	67434016	1.193877551	0.734693878	0.104162559
rs9603697	13	40783323	-0.253731343	0.559701493	0.650308734
rs962796	13	54385284	1.078571429	0.614285714	0.079120181
rs9818122	3	85861064	-0.219298246	0.399122807	0.582696292
rs9826775	3	156295341	0.270967742	0.638709677	0.671389024
rs9827823	3	84221774	-0.576923077	0.56043956	0.303286223
rs9888533	13	107854612	0.424	0.608	0.485572259
rs9926784	16	19941968	0.021097046	0.375527426	0.955198593
rs9944219	15	46500612	0.420168067	0.613445378	0.493387143
rs994596	4	18459828	0.853846154	0.584615385	0.144145481
rs9951619	18	56882326	0.480263158	0.559210526	0.390437884
